# Periodontal disease and cancer: Epidemiologic studies and possible mechanisms

**DOI:** 10.1111/prd.12329

**Published:** 2020-05-08

**Authors:** Ngozi Nwizu, Jean Wactawski‐Wende, Robert J. Genco

**Affiliations:** ^1^ Department of Diagnostic and Biomedical Sciences School of Dentistry The University of Texas Health Science Center at Houston Houston USA; ^2^ School of Public Health and Health Professions University at Buffalo, The State University of New York Buffalo USA; ^3^ Department of Cancer Prevention and Control Roswell Park Cancer Institute Buffalo USA; ^4^ Department of Oral Biology University at Buffalo, The State University of New York Buffalo USA

**Keywords:** cancer risk, epidemiology, inflammation, missing teeth, periodontal disease, tooth loss

## Abstract

Epidemiologic and cancer control studies on the association of periodontal disease and cancer risk mostly suggest a positive association with overall cancer risk and certain specific types of cancer. These findings are generally consistent among cross‐sectional and longitudinal studies. In this paper, we review epidemiologic studies and current knowledge on periodontal disease and cancer, with a focus on those studies conducted in the years following the Joint European Federation of Periodontology/American Academy of Periodontology Workshop on “Periodontitis and Systemic Diseases” in November 2012. This review also explores the role of chronic inflammation as a biologically plausible mechanistic link between periodontal disease and risk of cancer. Furthermore, it highlights studies that have examined the potential importance of certain periodontal pathogens in this association.

## 1 INTRODUCTION

Periodontal disease is a prototype of a locally destructive, chronic, low‐grade inflammatory process, and has been linked to increased cancer risk in a variety of epidemiologic studies.^1^, ^2^, ^3^, ^4^, ^5^, ^6^ As a result, there have been concerted research efforts in recent years towards establishing if such a causal association exists. The public health impact could potentially be huge if intervention measures that aid in the prevention and management of periodontal disease could also help reduce the risk of developing cancer or its progression.

Prior to the Joint European Federation of Periodontology/American Academy of Periodontology Workshop on “Periodontitis and Systemic Diseases” in November 2012, epidemiologic research on the association between periodontal disease and cancer mainly comprised observational studies and systematic reviews. It was determined at the workshop that although there were suggestions of a positive link between periodontal disease and cancer, particularly oral and oropharyngeal cancers, it was far too premature to infer the association was causal. Conveners of the workshop recommended that in order to lend credence to the existence of a causal association between periodontal disease and cancer, future studies should aim to fulfill the Bradford Hill or equivalent criteria. Such studies should employ precise and community‐agreed case definitions of periodontal disease states and avoid the use of surrogate markers. Furthermore, investigators were tasked with providing additional evidence that could support biological plausibility.^7^

Since that Joint European Federation of Periodontology/American Academy of Periodontology Workshop in November 2012, several more epidemiologic studies have been published on the relationship between periodontal disease and cancer risk, including a few large prospective studies, and several systematic reviews and meta‐analyses.^4^, ^5^, ^6^, ^8^, ^9^, ^10^, ^11^, ^12^, ^13^, ^14^ Conversely, there have been few intervention studies but no randomized controlled clinical trials.

In spite of the introduction of the standardized Center for Disease Control/American Academy of Periodontology periodontal disease clinical case definition for use in epidemiologic surveillance studies,^15^ many newer epidemiologic studies rely on proxy measures for periodontal disease ascertainment. Use of standardized clinical measures can be costly and labor‐intensive, especially in large surveillance studies. Consequently, a lack of uniformity in periodontal disease case definitions across epidemiologic studies persists. In addition, differences in study populations, sampling strategies, study designs, and methodologies make comparisons across studies challenging. It is worth noting, however, that newer epidemiologic studies are increasingly exploring alternative exposure measures, such as the oral microbiome, and specific groups of established periodontal pathogens, in an attempt to further understand the potential role of periodontal disease in cancer development.

This review provides a comprehensive assessment of existing epidemiologic evidence on the association between periodontal disease and cancer risk, with emphasis on research findings published after the November 2012 workshop on “Periodontitis and Systemic Diseases” until January 2018. Plausible mechanistic links between periodontal disease and cancer risk are discussed, as well as important knowledge gaps which might inform future directions for research and clinical applications.

## 2 PERIODONTAL DISEASE AND TOTAL CANCER RISK

Few published epidemiologic studies on periodontal disease and incident total cancer exist and these mostly point to a positive association. See Tables 1 and 2 for details. One early study from 2008 included 48 375 US male health professionals who were followed for an average of 17.7 years. The authors reported a statistically significant increased risk of total cancer among those with a history of periodontal disease (hazard ratio 1.14, 95% confidence interval 1.07‐1.22) and individual cancer sites of the lung, kidney, pancreas, and hematological system.^1^ However, the positive associations remained only for overall cancer risk and hematological cancers when restricted to never‐smokers. In a population‐based cohort of 15 333 Swedish twins, investigators found an increased overall cancer risk (hazard ratio 1.15, 95% confidence interval 1.01‐1.32) among participants with self‐reported tooth mobility involving at least half of their dentition after adjusting for established risk factors including smoking.^2^ Statistically significant associations were also observed for digestive tract, colorectal, pancreatic, prostate, and corpus uteri cancers, and also in individuals aged 51 years or older. In those reporting fewer loose teeth or who were less than 51 years of age, the associations between periodontal disease and cancers were attenuated. A large case‐control Japanese study that examined cancer risks at 14 different body sites found tooth loss was positively associated with risk of head and neck, esophageal, and lung cancers, although no association for lung cancer was found in never‐smokers.^3^

**Table 1:** Periodontal disease and total (incident) cancer estimates

Author(s), year, ref. no.	Research design	Study participants N (M/F)	Periodontal disease measure	Cancer cases N	Total cancer MV adjusted HR (95% CI)
Michaud et al, 2008^1^	Cohort (United States) HPFS	48 375 (M)	History of periodontal disease with bone loss	5720	1.14 (1.07‐1.22) Significant associations observed for cancers of lung, kidney, and pancreas, and hematological cancers
			Tooth loss 17‐24 teeth 0‐16 teeth Reference group: 25‐32 teeth	5720	0.95 (0.88‐1.02) 1.09 (0.99‐1.20)
Arora et al, 2010^2^	Cohort (Sweden) Swedish twins	15 333 (M/F)	History of tooth mobility	4361	1.15 (1.01‐1.32) Increased risks noted for cancers of the corpus uterine, colorectum, pancreas, and prostate
Hiraki et al, 2008^3^	Case‐control (Japan)	15 720 (M/F)	Tooth loss using a self‐administered questionnaire	5240	Overall cancer risk not assessed. Significant positive associations for risk of head and neck, esophageal, and lung cancers
Wen et al, 2014^8^	Cohort (Taiwan)	153 566 (M/F)	Evidence of periodontitis or gingivitis via insurance claims database. Reference group: individuals with gingivitis	3594	1.05 (1.00‐1.11) Higher risk of developing oral cancer
Nwizu et al, 2017^4^	Cohort (United States) WHI‐OS Study	65 869 (F)	Self‐reported history of periodontal disease	7149	1.14 (1.08‐1.20) Positive associations recorded for breast, lung, esophagus, gall bladder, and melanoma skin cancers
Michaud et al, 2016^5^	Cohort (United States) HPFS Study	19 933 (M)	Self‐reported history of periodontal disease	2959	1.13 (1.01‐1.27) 2.5‐fold increased risk in smoking‐related cancers (lung, bladder, oropharyngeal, esophageal, kidney, stomach, and liver cancers) seen in those with advanced periodontitis (data based on never‐smokers only)
Michaud et al, 2018^6^	Cohort (United States) ARIC Study	7466 (M/F)	CALs and a combination of CAL and PD, based on the CDC/AAP standardized clinical case definition	1648	1.24 (1.07‐1.44) For severe periodontitis vs no/mild periodontitis. Increased risks also noted for lung and colorectal cancers

Abbreviations: ARIC, Atherosclerosis Risk in Communities study; CAL, clinical attachment level; CDC/AAP, Center for Disease Control/American Academy of Periodontology; HPFS, Health Professional Follow‐Up Study; M/F, Male/Female; MV adjusted HR (95% CI), multivariate‐adjusted model hazard ratio (95% confidence interval); N, number of valid responses; PD, probing depth; ref. no., reference citation number; WHI‐OS, Women's Health Initiative Observational Study.

**Table 2:** Periodontal disease and total (mortality) cancer estimates

Author(s), year, ref. no.	Research design	Study participants N (M/F)	Periodontal disease measure	Cancer deaths N	Total cancer MV adjusted HR (95% CI)
Hujoel et al, 2003^16^	Cohort (United States) NHANES I epidemiologic follow‐up study	11 328 (M/F)	Periodontitis measured by Russell Index	714	1.55 (1.25‐1.92) Associated with increased risk of lung cancer mortality
Tu et al, 2007^17^	Cohort (Scotland, United Kingdom) Glasgow alumni	12 223 (M/F)	Tooth loss as oral health status index	549	1.00 (0.98‐1.02)
Cabrera et al, 2005^18^	Cohort (Sweden)	1462 (F)	Tooth loss assessed by: questionnaire and dental examination (panoramic radiographic survey)	68	1.18 (0.91‐1.52)
Aida et al, 2011^19^	Cohort (Japan)	4425 (M/F)	Oral health (tooth loss) via self‐administered questionnaires 19 or fewer teeth and eat everything 19 or fewer teeth and eating difficulty Reference group: (20 or more remaining teeth)	159	1.48 (0.39‐5.56) 1.40 (0.52‐3.79)
Abnet et al, 2005^20^	Cohort (China) The Nutrition Intervention Trial General Population Trial	29 584 (M/F)	Tooth loss via oral exam	3139	Overall risk of cancer risk not assessed Increased risk of upper GI cancer deaths
Heikkila et al, 2018^10^	Cohort (Finland)	68 273 (M/F)	Procedure codes for periodontal treatment recorded during dental visits	797	1.33, 1.10‐1.58 Higher pancreatic cancer mortality also seen

Abbreviations: GI, gastrointestinal; M/F, male/female; MV adjusted HR (95% CI), multivariate‐adjusted model hazard ratio (95% confidence interval); N, number of valid responses; NHANES, National Health and Nutrition Examination Survey; ref. no., reference citation number.

The earliest study examining the relationship between periodontal disease and cancer mortality was the US population‐based National Health and Nutrition Examination Survey I Epidemiologic Follow‐up Study. Standardized dental exams (Russell Index) characterized the presence of periodontal disease. Cancer mortality was positively associated with periodontitis at baseline exam (hazard ratio 1.55, 95% confidence interval 1.25‐1.92), especially for lung cancer mortality (hazard ratio 1.94, 95% confidence interval 1.16‐3.26). When stratified by smoking, periodontitis was associated with lung cancer in smokers (hazard ratio 1.94, 95% confidence interval 1.14‐3.30), but not in never‐smokers (hazard ratio 0.58, 95% confidence interval 0.12‐2.78).^16^

By contrast, findings from the Glasgow alumni cohort study found no association between the number of missing teeth and overall cancer mortality (hazard ratio 1.00, 95% confidence interval 0.98‐1.02) or lung cancer mortality.^17^ Two other studies reported no associations between the number of missing teeth (used as a proxy measure for periodontal disease) and cancer mortality among Swedish women and an older Japanese population, respectively.^18^, ^19^ Abnet et al^20^ found a positive relationship between greater tooth loss and upper gastrointestinal cancer mortality (irrespective of smoking status), but not for mortality of other cancer sites. In many of these studies, assessment of periodontal disease was either through self‐report or tooth loss status, which potentially introduced misclassification. Also, using missing teeth as a proxy for periodontal disease would introduce misclassification if tooth loss was attributed to caries or other causes. In addition, inadequate information and control for confounding by smoking may account for observed differences across studies. Case‐control studies make the establishment of temporality difficult. Lastly, differences in age, gender, and use of twin populations may limit generalizability.

### 2.1 Newer epidemiologic evidence

A study involving a large Taiwanese cohort reported a modest risk of cancer overall and periodontal disease after adjusting for potential confounders (hazard ratio 1.05, 95% confidence interval 1.00‐1.11).^8^ However, they used a relatively young cohort, and individuals with gingivitis served as their reference group, potentially limiting their ability to detect a strong association. Michaud et al^9^ conducted a systematic review and meta‐analysis on periodontal disease, tooth loss, and cancer risk based on 50 studies from 46 publications up until July 2016. Periodontal disease status was ascertained variously via oral exams, dental records, radiographs, self‐report, and national health insurance data. A total of 13 cohort and five case‐control studies examined the association between periodontal disease and risk of cancer, while 14 cohort and 20 case‐control studies explored the association between tooth loss (a proxy measure for periodontal disease) and cancer risk. A positive association between periodontal disease and total cancer risk was noted in five cohort studies. However, only three of these studies included smoking in their multivariate models and their total cancer risk estimates ranged from 14% to 20% among those with periodontal disease. Positive associations were also reported for oral, lung, and pancreatic cancers.

There have been at least three original published studies involving large cohorts within the last 3 years and these support a positive relationship between periodontal disease and increased cancer risk.^4^, ^5^, ^6^ They include a prospective study of 65 869 postmenopausal women from the Women's Health Initiative‐Observational Study that found self‐reported history of periodontal/gum disease was associated with a 14% statistically increased risk of total cancer (multivariate‐adjusted model hazard ratio 1.14, 95% confidence interval 1.08‐1.20) and positive associations for esophageal, breast, lung, gall bladder, and melanoma skin cancers.^4^ A validation of self‐report in a subset of these women using dental records suggested possible underreporting of periodontal disease,^21^ which may have weakened the observed association. Nwizu et al^4^ also reported a positive association among never‐smokers in their study (multivariate‐adjusted model hazard ratio 1.12, 95% confidence interval 1.04‐1.22), supporting positive associations observed among a male‐only cohort of never‐smokers (n = 19 933) from the Health Professionals Follow‐up Study. They found self‐reported periodontal disease was associated with a 13% increased risk of total cancer (multivariate‐adjusted model hazard ratio 1.13, 95% confidence interval 1.01‐1.27). The risk (45%) was more pronounced in men with advanced periodontitis (those with <17 remaining teeth).^5^

The third study involved an elderly cohort of 7466 participants from the Atherosclerosis Risk in Communities study,^6^ which provides some of the most compelling epidemiologic evidence to date. Periodontal disease severity was ascertained via self‐report of edentulism, or by a comprehensive dental exam (probing depth and gingival recession measurements at six sites on all teeth) for those with teeth. Based on the Center for Disease Control/American Academy of Periodontology standardized clinical case definition for surveillance studies,^15^ clinical attachment level, and a combination of clinical attachment level and probing depth, were determined. The authors controlled for a number of possible confounders including smoking dose and duration. Participants who had severe periodontitis (>30% of sites with an attachment loss of >3 mm) had a 24% elevated total cancer risk when compared with those with no/mild periodontitis (<10% of sites with an attachment loss of >3 mm) (hazard ratio 1.24, 95% confidence interval 1.07‐1.44, *P*_trend_ = .004). Slightly higher risks (28%) were reported for edentulous participants relative to those with no or mild periodontitis. Similar positive but statistically insignificant results were observed among never‐smokers and in participants classified as having severe periodontitis, based on the Center for Disease Control/American Academy of Periodontology definition. Participants with periodontal disease were especially vulnerable to lung, colorectal, and overall cancer. White participants with periodontal disease appeared more at risk than their black counterparts.

Another more recent publication evaluated the relationship between periodontal disease and cancer mortality in a registry‐based cohort of 68 273 Helsinki adults followed over a 10‐year period.^10^ Periodontal disease was defined using procedure codes for periodontal treatment recorded during dental visits. Periodontitis was associated with higher cancer mortality overall (relative risk 1.33, 95% confidence interval 1.10‐1.58), and especially for pancreatic cancer mortality (relative risk 1.69, 95% confidence interval 1.04‐2.76). The major drawback to this study was the lack of adjustment for smoking. Curiously, the authors found no association between periodontal disease and lung cancer mortality.

The evidence provided through more recent well‐designed prospective cohort studies support an association of periodontal disease and overall cancer risk, and risk of certain specific cancer sites, including cancers of the lung and upper digestive tract.

## 3 PERIODONTAL DISEASE AND HEAD AND NECK CANCER RISK

Risk of head and neck cancers, especially those of the oral cavity and oropharynx, have been the most extensively explored in relation to periodontal disease‐cancer associations. The majority of these studies report a positive association, including those from across the world.^22^, ^23^, ^24^, ^25^, ^26^, ^27^, ^28^, ^29^, ^30^, ^31^, ^32^, ^33^, ^34^, ^35^, ^36^, ^37^, ^38^ Periodontal disease has been positively linked to oral cancer in particular in studies conducted in the USA,^22^ Europe,^31^ Latin America,^33^ India,^35^ China,^23^ and Brazil,^28^ and also to a precancerous oral lesion, leukoplakia.^24^, ^32^

Wide variations in case definitions and measures of periodontal disease have been used, yet yield similar findings. Frequently, one or more surrogate measures of periodontal disease were used, including self‐reported history of periodontal disease via structured questionnaires or interviews,^24^, ^27^, ^28^, ^38^ or assessment of tooth loss or poor dentition.^22^, ^23^, ^24^, ^26^, ^27^, ^29^, ^30^, ^31^, ^33^, ^38^, ^39^ Use of an oral clinical examination,^40^ or radiographic assessments for alveolar bone loss,^34^, ^37^, ^41^ were much less frequent, although a number of studies combined both approaches.^22^, ^23^, ^30^, ^31^, ^32^, ^35^, ^36^

One study utilized 13 798 adults from the National Health and Nutrition Examination Survey III Study. Mean clinical attachment loss (≥1.5 mm), used as a measure of periodontal disease severity, was associated with an increased overall risk of oral tumors (odds ratio 4.57, 95% confidence interval 2.25‐9.30) after adjusting for many confounders including smoking and alcohol consumption.^32^ Rezende et al^36^ assessed periodontal disease severity by a self‐reported questionnaire and an oral exam, and categorized advanced disease as two sites with clinical attachment level of ≥6 mm and probing pocket depth of ≥7 mm at proximal sites of two different teeth. In their study of 35 cases and 40 controls, a positive association between advanced periodontal disease and oral cancer was found, regardless of the oral hygiene and dental status of the participants. Another case‐control study involving 200 oral/oropharyngeal cancer cases and 200 controls reported a statistically significant association between the number of missing teeth and cases of oral/oropharyngeal cancer (≥16 teeth; odds ratio 2.74, 95% confidence interval 1.23‐6.12).^30^

By contrast, a few studies have reported mixed or negative results. Investigators of a Carolina population‐based, case‐control study examined 1361 cases and 1289 frequency‐matched controls and found self‐reported history of tooth mobility was positively associated with head and neck squamous cell carcinoma (odds ratio 1.33, 95% confidence interval 1.07‐1.65) after controlling for possible confounders. However, this association was nonsignificant in never‐smokers. They also reported that tooth loss (16‐28 vs 0‐5 lost teeth) was not associated with squamous cell carcinoma of the head and neck risk.^38^ Additionally, a large cohort study did not observe any statistically meaningful relationships between periodontal disease and oropharyngeal cancers among their adult male‐only participants (hazard ratio 1.15, 95% confidence interval 0.73‐1.81).^1^

### 3.1 Newer epidemiologic evidence

Research evidence from recent years repeatedly indicates that periodontal disease is linked to an increased risk of head and neck cancer. In 2013, three different meta‐analyses were conducted into this association. One of the meta‐analyses examined periodontal disease and risk of head and neck cancer.^42^ The other two evaluated tooth loss with head and neck cancer risk.^43^, ^44^ The meta‐analysis by Zeng et al^42^ relied on findings from two cohort studies and six case‐control studies. Different periodontal disease measures were used and included alveolar bone loss (three studies), clinical attachment loss (one study), Community Periodontal Index of Treatment Needs (one study), tooth mobility (one study), and poor oral condition (one study). Results from the meta‐analysis revealed a statistically significant association between periodontal disease and head and neck cancer risk (odds ratio 2.63, 95% confidence interval 1.68‐4.14). In the meta‐analysis by Wang et al^43^ involving eight case‐control studies and one cross‐sectional study (n* *=* *5204 cases, n =* *5518 controls), tooth loss was significantly associated with head and neck cancer (odds ratio 2.00, 95% confidence interval 1.28‐3.14). Head and neck cancer risk remained strong among those with moderate (18%) and severe (54%) tooth loss, respectively. The third meta‐analysis, also by Zeng et al,^44^ involved one cohort study and 10 case‐control studies. They reported a significantly increased risk of head and neck cancer (odds ratio 1.58, 95% confidence interval 1.08‐2.32) among participants who had lost 6‐15 teeth. The risks were progressively higher among those with a loss of more than 15 teeth.

Similarly, findings from other, more recent meta‐analyses^45^, ^46^ and one systematic review^47^ all point to an association between periodontal disease and oral cancer risk. In a meta‐regression analysis involving seven case‐control studies, the authors observed a linear‐dose response between the number of missing teeth and oral cancer risk. For each additional missing tooth, the odds ratio significantly increased by 0.03 (95% confidence interval 0.01‐0.05), although moderate heterogeneity (*I* ^2^ = 67.5%, *P *=* *0.003) was present.^9^ There was, however, no linear‐dose effect observed with respect to head and neck cancer risk.

A retrospective study comprising 178 oral cancer cases and 123 controls examined the relationship between chronic periodontitis (evidenced by bone loss) and oral squamous cell carcinoma. Higher mean bone loss was related to an increased oral cancer risk (odds ratio 2.4, 95% confidence interval 1.5‐3.8).^14^ Similar positive associations were reported in other case‐control studies between history of periodontal disease and head and neck cancer risk,^48^ and generalized gingival recession in relation to oral cancer risk.^49^ Those study findings are, however, in contradiction to a case‐control study among the Han Chinese population that found no significant association between tooth loss and risk of oral cancer.^50^

Other published epidemiologic studies include two large prospective studies. A Taiwanese‐based cohort study (n* *=* *148 166) reported a positive association between chronic periodontitis and oral cancer (hazard ratio 1.20, 95% confidence interval 1.09‐1.33).^11^ Of note, patients with gingivitis served as the reference group. The second cohort study, of elderly US women (n = 65 869), did not find any association between self‐reported periodontal disease and cancers of the lips, oral cavity, and pharynx combined (hazard ratio 1.10, 95% confidence interval 0.64‐1.87).^4^

Head and neck cancers comprise a heterogeneous group of conditions with varying risk factors. Clinical periodontal measures, proxy measures of periodontal disease, or a combination of both methods, have been used in evaluating its associations with head and neck cancers. Many of the studies controlled for smoking and alcohol consumption, but not all of them. Causality was difficult to ascertain as the majority were case‐control studies. Many studies involved small sample sizes. Even in larger cohorts, the numbers of head and neck cancer cases were few because it is not a common cancer. For example, only 118 cases of oropharyngeal cancer occurred in 48 375 participants in the Health Professionals Follow‐Up Study.^1^ A total of 68 cases of lip, oral cavity, and pharynx cancers occurred among 65 869 women participants in the Women's Health Initiative Observational Study.^4^ In summary, the existing body of literature points to a positive association between periodontal disease and head and neck cancer risk.

## 4 PERIODONTAL DISEASE AND RISK OF CANCERS OF THE DIGESTIVE TRACT

A number of studies have examined the association between periodontal disease and cancers of the digestive tract. Results from early studies indicate periodontal disease is linked to an increased risk of upper gastrointestinal tract cancer incidence and mortality.^51^, ^52^, ^53^, ^54^ A study of healthy, rural Chinese adults (n =* *29 584) reported higher tooth loss was significantly associated with risk of upper gastrointestinal cancer mortality (n = 2625 deaths, hazard ratio 1.35, 95% confidence interval 1.14‐1.59), and this increase was not limited to smokers alone. Assessment of tooth loss measured via oral examination was based on greater than age‐specific median number of teeth lost.^20^ Males who had never smoked had higher hazard ratios (hazard ratio 1.59, 95% confidence interval 1.03‐2.45) than those with a history of smoking (hazard ratio 1.39, 95% confidence interval 1.06‐1.83). There was no statistical association in females (hazard ratio 1.24, 95% confidence interval 0.98‐1.58). Based on results from the subgroup analyses that assessed the impact of smoking, the authors speculated that smoking could not have accounted for the effects observed. A National Health and Nutrition Examination Survey III study consisting of 12 605 men and women revealed orodigestive cancer mortality risk was strongly associated with moderate/severe periodontitis, and that risk increased with severity of periodontal disease (relative risk 2.28, 95% confidence interval 1.17‐4.45; *P*_trend_ = .01).^55^ Higher periodontitis‐associated mortality rates were also observed in individuals with colorectal or pancreatic cancers. This study utilized qualified examiners for the standardized oral exams, including clinical attachment level and periodontal pocket depth measurements. However, the number of deaths from orodigestive cancer based on counts from individual subsites within the region was small (n* *=* *157).

A more recent study involving elderly Japanese found a positive association between tooth loss and orodigestive cancer mortality.^56^ The latest study on periodontal disease and orodigestive cancer risk relied on clinical measures based on the Center for Disease Control/American Academy of Periodontology definition and found no association between mild, moderate, or severe periodontitis relative to risk of orodigestive cancer.^6^ Individuals with no evidence of periodontitis were the reference group. No differences in risk were observed for race or in never‐smokers.

The majority of evidence for periodontal disease and orodigestive/upper gastrointestinal cancer suggests a positive association, but most of the data are based on mortality rather than incidence.

## 5 PERIODONTAL DISEASE AND RISK OF CANCERS OF THE DIGESTIVE TRACT – ESOPHAGEAL CANCER

Studies assessing the risk of esophageal cancers have been largely population‐based involving participants primarily from China,^51^, ^57^, ^58^ Iran,^59^, ^60^ and the Nordic countries.^52^, ^61^ Tooth loss measures were often the mode to ascertain periodontal disease status.^3^, ^51^, ^52^ The majority of findings suggest a higher risk of esophageal cancers in patients with existing periodontal disease.^3^, ^51^, ^57^, ^59^ The drawback is that the number of participants in most of these studies was small and proxy measures of periodontal disease were often used. In one of the studies based in China, Abnet et al^51^ found esophageal cancer was positively associated with tooth loss (relative risk 1.3, 95% confidence interval 1.1‐1.6). However, a similar study set in Finland did not find any such association with esophageal cancers (squamous cell carcinoma or adenocarcinoma subtypes).^52^ A much larger nested case‐control Swedish study examined 6156 esophageal squamous cell carcinomas, 2684 esophageal adenocarcinomas, and 38 308 gastric cancer cases compared with 29 993, 15 036, and 99 991 controls, respectively.^61^ An increased risk of esophageal adenocarcinoma (odds ratio 1.7, 95% confidence interval 1.1‐2.6) was found, but not for esophageal squamous cell carcinoma or gastric cancer. The study did not discriminate between different forms of oral disease including periodontal disease, and evidence of hospitalization for chronic obstructive pulmonary disease was used as a surrogate marker for smoking status. As such, comparison with other studies which had better, more direct measures of smoking or periodontal disease status is difficult. A large case‐control study in Japan (cases, n =* *5240; controls, n =* *10 480), found an increased risk of esophageal cancers among participants who had 1‐8 remaining teeth (odds ratio 2.36, 95% confidence interval 1.17‐4.75; *P*_trend_ = .002) compared with those with ≥21 remaining teeth. The risk of esophageal cancer was even stronger among never‐smokers (odds ratio 9.50, 95% confidence interval 1.33‐67.98; *P*_trend_ = .021), but absent among former or current smokers.^3^ The wide confidence intervals observed indicate small numbers of esophageal cancers among never‐smokers and less certainty. Also, tooth loss was measured following cancer diagnosis so reverse causation is possible. In a large, male‐only, US cohort study, a positive, near‐significant association for esophageal cancer was observed after adjusting for potential confounders including smoking (hazard ratio 1.44, 95% confidence interval 0.98‐2.11).^1^

### 5.1 Newer epidemiologic evidence

More recent studies reporting on the association between periodontal disease and esophageal cancer risk indicate a strongly positive association. These include a total of four meta‐analyses published during year 2015‐2017.^9^, ^62^, ^63^, ^64^ Results from pooled analyses of one meta‐analysis showed an elevated risk of esophageal cancer (odds ratio 1.36, 95% confidence interval 1.16‐1.59; *I* ^2^ = 0) among those with the highest loss of teeth compared with those with the lowest loss of teeth. Their meta‐analysis comprised eight studies (cohort, n = 3; case‐control, n = 5), all of which adjusted for multiple confounders including smoking.^62^ The authors stated they observed no evidence of heterogeneity or publication bias. Wang et al^63^ based their meta‐analysis on three cohort studies and six case‐control studies, and reported a combined odds ratio of 1.53 (95% confidence interval 1.02‐2.29) for esophageal cancer in those with significant tooth loss. A statistically significant dose‐response effect was present (summary odds ratio for each increasing tooth loss 1.01, 95% confidence interval 1.00‐1.02). In the third meta‐analysis, which was drawn from three cohort studies, five case‐control studies, and one cross‐sectional study, tooth loss was also positively linked to an increased risk of esophageal cancer in their pooled analysis (relative risk 1.30, 95% confidence interval 1.06‐1.60, *I* ^2^ = 13.5%). A significant dose‐response relationship was present (relative risk 1.01, 95% confidence interval 1.00‐1.03; *P* for nonlinearity = .45).^64^ In these latter two meta‐analyses, inconsistencies were noted across studies and may limit generalizability. However, the last meta‐analysis, of three cohort studies and four case‐control studies, reported a lack of dose‐response between tooth loss and esophageal risk.^9^

A 3‐fold higher risk of esophageal cancer was observed among participants in the Women's Health Initiative‐Observational Study reporting history of periodontal disease after controlling for relevant confounders, including pack‐years of smoking (hazard ratio 3.28, 95% confidence interval 1.64‐6.53). A statistically significant risk of esophageal and gastric cancers combined was also noted among never‐smokers (hazard ratio 2.26, 95% confidence interval 1.19‐4.29).^4^ Stratified analyses on smoking in esophageal carcinoma cases alone could not be performed because there were too few cases (n =* *34). These findings are similar to those of a follow‐up study involving never‐smokers in a large, US, male‐only, cohort study, in which a statistically significant increased risk in esophageal and oropharyngeal cancers combined with periodontal disease was found (hazard ratio 2.25, 95% confidence interval 1.30‐3.90).^5^ Lastly, Chen et al^65^ reported an increased risk of tooth loss with the esophageal squamous cell carcinoma subtype; however, this did not reach statistical importance (odds ratio 1.29, 95% confidence interval 0.94‐1.74). Higher risk was observed with increasing numbers of tooth loss (>6 teeth lost vs none, odds ratio 1.48, 95% confidence interval 1.04‐2.11).

Based on the available evidence, especially from newer epidemiologic studies, there are strong indications that periodontal disease is positively linked to esophageal cancers. Some of these studies involved large, prospective cohorts, although the number of esophageal cancers was still small. The risk persisted when limited to never‐smokers, eliminating potential confounding by smoking as a basis for the observed association. Differences across regions and esophageal cancer subtypes may account for some of the variations observed across studies.

## 6 PERIODONTAL DISEASE AND RISK OF CANCERS OF THE DIGESTIVE TRACT – GASTRIC CANCER

There has been at least one study linking periodontal disease with an increased risk of premalignant gastric lesions,^66^ and early research evidence regarding the relationship between periodontal disease and gastric cancers has been largely mixed, although it mostly points towards a lack of association. This evidence includes two cohort and two case‐control studies, all of which reported no association. The authors examined associations between risk of gastric cancer and different periodontal disease proxy measures, which included self‐report (US cohort, hazard ratio 1.13, 95% confidence interval 0.72‐1.79),^1^ tooth mobility (Swedish cohort, hazard ratio 0.85, 95% confidence interval 0.45‐1.59),^2^ tooth loss (Japanese population, odds ratio 0.90, 95% confidence interval 0.58‐1.41),^3^ and oral diseases that also included periodontal disease (Swedish adults, odds ratio 0.9, 95% confidence interval 0.7‐1.1).^61^ By contrast, while a Chinese‐based study associated tooth loss with increased risk of both gastric cardia (relative risk 1.3, 95% confidence interval 1.0‐1.6) and gastric noncardia cancers (relative risk 1.8, 95% confidence interval 1.1‐3.0),^51^ another study involving a Finnish cohort showed a positive link between tooth loss and gastric noncardia cancer (relative risk 1.65, 95% confidence interval 1.09‐2.49), but not gastric cardia adenocarcinoma.^52^ Associations with gastric cancer mortality are largely negative, based on information from study populations in the USA (odds ratio 0.95, 95% confidence interval 0.39‐2.35;^16^ relative risk 0.99, 95% confidence interval 0.38‐2.58^55^) and Japan (hazard risk 1.09, 95% confidence interval 0.89‐1.22).^56^

### 6.1 Newer epidemiologic evidence

Epidemiologic literature from more recent years on the association between periodontal disease and gastric cancer also shows varied results. A meta‐analysis conducted in 2016 based on combined findings from five cohort and four case‐control studies concluded that tooth loss may be linked to gastric cancer (cohort studies combined relative risk 1.31, 95% confidence interval 1.12‐1.53; case‐control studies combined relative risk 1.86, 95% confidence interval 1.08‐3.21). This was based on comparisons between the highest vs lowest categories of missing teeth.^67^ However, a case‐control study from Iran found subcategories of tooth loss were not associated with risk of gastric adenocarcinomas overall, and its histologic subtypes, gastric cardia adenocarcinoma, and gastric noncardia adenocarcinoma. The only exception was where the loss of many teeth (25‐31 teeth) was associated with a significantly increased risk of the gastric cardia adenocarcinoma subtype (odds ratio 3.5, 95% confidence interval 1.2‐9.7). In this study, a loss of ≤12 teeth was used as the reference group.^68^ Similarly, a more recent USA‐based cohort study of older women observed no such association (hazard ratio 1.58, 95% confidence interval 0.94‐2.67).^4^

Given all of the available evidence, the relationship (if any) between periodontal disease and gastric cancers is unclear. Many of the studies controlled for important confounding factors. However, most of these studies were case‐control studies which relied on proxy measures of periodontal disease.

## 7 PERIODONTAL DISEASE AND RISK OF CANCERS OF THE DIGESTIVE TRACT – PANCREATIC CANCER

Some early epidemiologic studies have demonstrated a positive relationship between periodontal disease and pancreatic cancer. These include a study of 29 104 male Finnish smokers from the Alpha‐Tocopherol, Beta‐Carotene Cancer Prevention cohort study, where loss of dentition (edentulous compared with the loss of 0‐10 teeth) was used as a proxy measure for periodontal disease (hazard ratio 1.63, 95% confidence interval 1.09‐2.46, *P*_trend_ = .02).^69^ Similar positive associations were observed among a Swedish twin cohort of 15 333 individuals (hazard ratio 2.06, 95% confidence interval 1.14‐3.75)^2^ and a US cohort of 51 529 male health professionals (relative risk 1.64, 95% confidence interval 1.19‐2.26).^70^ An increased risk of pancreatic cancer was also observed in never‐smokers (relative risk 2.09, 95% confidence interval 1.18‐3.71) in the latter study. The investigators indicated that timing and severity of periodontal disease were associated with the greatest risk of developing pancreatic cancer (relative risk 2.70, 95% confidence interval 1.70‐4.32). However, neither the number of natural teeth at baseline, nor the cumulative tooth loss during follow‐up, was significantly linked with pancreatic cancer risk. All of these studies were prospective in nature and controlled for smoking and other pertinent confounding factors, with the exception of the study by Stolzenberg‐Solomon et al,^69^ which only involved smokers. A large, hospital‐based, case‐control study found only a modest positive association in the relationship between pancreatic cancer risk and the presence of oral disease (odds ratio 1.2, 95% confidence interval 1.0‐1.4).^71^ Study participants were limited to those with evidence of oral disease at least 5 years previously, to limit the influence of reverse causality. However, because they did not differentiate periodontal disease from other oral conditions, it is difficult to assess the association between periodontal disease and pancreatic cancer. Hiraki et al^3^ found no association between periodontal disease and risk of pancreatic cancer in their case‐control study of 15 720 participants with 178 pancreatic cancer cases. While a National Health and Nutrition Examination Survey I study did not find a link between clinical measurements of periodontal disease and pancreatic cancer mortality (odds ratio 1.77, 95% confidence interval 0.85‐3.67),^16^ a National Health and Nutrition Examination Survey III study reported a borderline association (relative risk 4.56, 95% confidence interval 0.93‐22.29).^55^

### 7.1 Newer epidemiologic evidence

Over the last few years, there have been several more publications concerning periodontal disease and pancreatic cancer risk. These studies mostly adjusted for smoking among many other potential confounders and reported results of their stratified analyses, or restricted analyses to never‐smokers in an attempt to minimize or eliminate the influence of smoking.

One study, which involved 214 890 Taiwanese individuals with 107 cases of pancreatic cancers, found an increased risk among those with recorded evidence of periodontal disease identified through their National Health Insurance Research Database (hazard ratio 1.55, 95% confidence interval 1.02‐2.33). Pancreatic cancer risk was more pronounced among those aged ≥65 years (hazard ratio 2.17, 95% confidence interval 1.03‐4.57), but lacking in those aged <65 years (hazard ratio 0.83, 95% confidence interval 0.52‐1.34).^72^ Unfortunately, history of smoking and alcohol consumption were not taken into account because of nonavailability of this data. They did, however, try to indirectly control for smoking by adjusting for conditions where chronic smoking and alcohol consumption are established risk factors, such as chronic obstructive pulmonary disease and alcoholic liver disease. A cohort study of 19 924 Swedish participants with 126 recorded cases of pancreatic cancer showed an increased but insignificant risk of pancreatic cancer among individuals with 0‐10 (hazard ratio 1.3, 95% confidence interval 0.7‐2.3) or 11‐20 teeth (hazard ratio 1.2, 95% confidence interval 0.7‐2.0) compared with those with ≥21 teeth, respectively.^73^ However, poor oral hygiene in individuals with <10 teeth was associated with an elevated risk of pancreatic cancer (hazard ratio 2.0, 95% confidence interval 1.0‐4.1) following adjustments for important confounding factors such as history of smoking and alcohol consumption. Periodontal disease status was determined by dental examination. Although the overall patient population was large, the number of patients with pancreatic cancer was small, thus limiting the ability of the authors to perform a more robust investigation of the effects of smoking and other important factors.

A meta‐analysis conducted in 2017 examined the evidence relating to periodontitis, or edentulism and pancreatic cancer risk.^74^ Eight studies were included in the meta‐analysis and consisted of clinical measures of periodontal disease (three studies) and surrogate measures including self‐reported history of periodontal disease, information from a health registry (three studies), or self‐reported tooth loss (two studies). Their findings support that periodontitis is related to pancreatic cancer risk (summary relative risk 1.74, 95% confidence interval 1.41‐2.15). The authors also reported finding no evidence of heterogeneity across studies and no publication bias. However, two of the studies did not control for smoking.

On the contrary, an updated analysis of male never‐smokers in the Health Professionals Follow‐up Study did not find an association between self‐reported periodontal disease and pancreatic cancer risk among their 141 cases of pancreatic cancer (hazard ratio 1.57, 95% confidence interval 0.98‐2.50).^5^ In the same manner, a prospective Women's Health Initiative‐Observational Study in older females did not report any association for pancreatic cancer overall (hazard ratio 0.89, 95% confidence interval 0.67‐1.18) or in never‐smokers (hazard ratio 0.89, 95% confidence interval 0.58‐1.35).^4^ A longitudinal, retrospective cohort study also failed to find a connection between periodontal disease and pancreatic cancer risk (hazard ratio 1.15, 95% confidence interval 0.75‐1.78).^8^ Participants with gingivitis served as their comparison cohort and this may have influenced the observed findings.

With respect to studies on pancreatic cancer mortality, a study in elderly Japanese (n =* *656) reported no association between periodontal disease and risk of pancreatic cancer mortality (hazard ratio 0.96, 95% confidence interval 0.83‐1.11).^56^ By contrast, a study (n =* *68 273) from Helsinki with 75 cases of pancreatic cancers reported observing a greater than 2‐fold increase in pancreatic cancer mortality risk. They did not, however, control for smoking and alcohol consumption.^10^

The precise nature of the association between periodontal disease and pancreatic cancer risk has yet to be fully determined. The role of smoking relative to this association has not been clearly delineated. However, the fact that periodontal disease has been positively linked to pancreatic cancer risk among never‐smokers in a few large prospective studies may be an indication that periodontal disease has a role in the development of pancreatic cancer independent of smoking status, and should be explored further.

## 8 PERIODONTAL DISEASE AND RISK OF CANCERS OF THE DIGESTIVE TRACT – COLORECTAL CANCER

Studies of the relationship between periodontal disease and colorectal cancer risk, although limited in number, mostly point to an absence of any association. The Health Professionals Follow‐up Study did not find any link between colorectal cancer risk and either self‐reported periodontal disease (hazard ratio 1.05, 95% confidence interval 0.90‐1.23) or tooth loss (0‐16 remaining teeth; hazard ratio 1.10, 95% confidence interval 0.87‐1.37) after adjustments for smoking and other important factors.^1^ The reference group for tooth loss comprised individuals with 25‐32 remaining teeth. A case‐control study among Japanese adults also found no significant association between varying degrees of edentulism (0, 1‐8, and 9‐20 remaining teeth) used as a surrogate measure for periodontal disease and colon cancer risk. Individuals with 21 remaining teeth served as the reference group.^3^

Using clinical measures of periodontal disease, a National Health and Nutrition Examination Survey I follow‐up study found no association with risk of colon cancer mortality (odds ratio 0.91, 95% confidence interval 0.49‐1.70),^16^ but a National Health and Nutrition Examination Survey III study showed a significantly elevated risk of death from colorectal cancer (relative risk 3.58, 95% confidence interval 1.15‐11.16).^55^ In these two studies, several factors including smoking were adjusted for in their multivariate analyses, but no statistical analysis was conducted to test for intra‐ and inter‐examiner variability, although the dental examiners were required to adhere to a strict protocol during their oral examination. The wide confidence interval observed with the estimates from the National Health and Nutrition Examination Survey III study are attributable to the small number of colorectal cancer cases.

The only study reporting a significantly elevated risk of colorectal cancer (62%) with periodontal disease involved a Swedish cohort of 15 333 twins (hazard ratio 1.62, 95% confidence interval 1.13‐2.33),^2^ which adjusted for individual level cancer risk factors and potential confounders. Self‐reported tooth mobility was used as a proxy measure for periodontal disease and that may have resulted in some degree of disease misclassification, either from the omission of mild cases of periodontal disease, or because of the inclusion of cases from other causes of tooth mobility besides periodontal disease.

### 8.1 Newer epidemiologic evidence

A meta‐analysis conducted in 2017 investigated the association between periodontal disease and colorectal cancer based on data obtained from four cohort studies.^9^ One study focused on risk of death from colorectal cancer rather than incident colorectal cancer risk. Three of the cohort studies ascertained periodontal disease status via clinical measures, while a fourth study used tooth mobility as a surrogate measure. Two studies had small samples sizes. The summary estimate for the meta‐analysis showed an elevated but statistically insignificant risk of colorectal cancer, with evidence of heterogeneity across studies (relative risk 1.47, 95% confidence interval 0.95‐2.29; *I* ^2^ = 66.5%, *P *=* *.03). Three subsequent studies also did not find a link with colorectal cancer. One of these studies included 7466 participants from the Atherosclerosis Risk in Communities study. Periodontitis status was based on the Center for Disease Control/American Academy of Periodontology definition. They determined mild, moderate, or severe periodontitis was not associated with colorectal cancer risk, even when limited to never‐smokers,^6^ although an increased risk of colorectal cancer was observed among participants with evidence of clinical attachment level or complete edentulism. The other two studies reported a similar lack of association among postmenopausal women from the Women's Health Initiative‐Observational Study (hazard ratio 0.98, 95% confidence interval 0.82‐1.17),^4^ and among never‐smokers in the Health Professionals Follow‐Up Study (hazard ratio 1.03, 95% confidence interval 0.75‐1.39.)^5^ In both studies, periodontal disease status was ascertained via self‐report.

In the Nurses’ Health Study (n* *=* *77 443) with 1165 documented cases of incident colorectal cancer, insignificant associations were found (hazard ratio 0.91, 95% confidence interval 0.74‐1.12), even when restricted to those with moderate to severe periodontal disease (hazard ratio 1.22, 95% confidence interval 0.91‐1.63).^75^ Tooth loss was also insignificantly associated with increased risk of incident colorectal cancer in women with <17 remaining teeth compared with those with 25‐32 teeth (hazard ratio 1.20, 95% confidence interval 0.91‐1.63). Similar positive associations were observed with respect to tooth loss and cancer risk involving the proximal colon and rectum, but not the distal colon.

Ren et al^76^ adopted a nested case‐control study based on two Chinese cohorts, the Shanghai Men's Health Study and the Shanghai Women's Health Study combined (cases, n* *=* *825; controls, n* *=* *3298), and a US cohort, the Southern Community Cohort Study (cases, n = 238; controls, n = 2258). They found no association between varying categories of tooth loss (1‐5, 6‐10, >10 teeth) and colorectal cancer risk, in either the Shanghai Men's Health Study/Shanghai Women's Health Study or Southern Community Cohort Study cohort.^76^ The investigators also conducted a meta‐analysis that incorporated findings from their own original study of three cohorts and those of three other older cohorts. The older cohort studies included a National Health and Nutrition Examination Survey I study that controlled for age and sex only,^16^ the Health Professionals Follow‐Up Study that adjusted for many pertinent factors including smoking and pack‐years of smoking history,^1^ and a National Health and Nutrition Examination Survey III study that investigated risk of colorectal cancer mortality and adjusted for smoking, age, gender, and socioeconomic factors.^55^ Two of these studies were based on clinical measures of periodontal disease, while the third was based on self‐reported history of periodontal disease. Results from their meta‐analysis provided further evidence that periodontal disease or tooth loss was not linked to colorectal cancer risk (hazard ratio 1.05, 95% confidence interval 0.86‐1.29).^76^ Finally, multivariate adjusted models that controlled for smoking among other factors in a cohort of 80‐year‐old, relatively healthy Japanese demonstrated no link between the number of teeth lost and the risk of colon cancer (hazard ratio 1.04, 95% confidence interval 0.92‐1.18).^56^ Existing data do not currently support an association between periodontal disease and colorectal cancer.

## 9 PERIODONTAL DISEASE AND LUNG CANCER RISK

Lung cancer risk in relation to periodontal disease has mostly been examined as part of larger studies exploring total cancer risk and other cancer‐specific subsites, with varying results. In the Health Professionals Follow‐Up Study there was an elevated lung cancer risk among individuals with a self‐reported history of periodontal disease (hazard ratio 1.36, 95% confidence interval 1.15‐1.60), but this association disappeared when restricted to never‐smokers (hazard ratio 0.96, 95% confidence interval 0.46‐1.98).^1^ Hiraki et al^3^ also reported increasing lung cancer risk with increasing numbers of missing teeth (odds ratio 1.54, 95% confidence interval 1.05‐2.27; *P*_trend_ = .027), but Arora et al^2^ did not find any association between advanced periodontal disease, characterized by the presence of tooth mobility involving at least 50% of the dentition, and lung cancer risk (hazard ratio 1.41, 95% confidence interval 0.81‐2.46).

In the National Health and Nutrition Examination Survey Epidemiologic Follow‐Up Study, lung cancer mortality demonstrated the strongest association with periodontal disease relative to other different cancer subtypes (odds ratio 1.94, 95% confidence interval 1.16‐3.26), following adjustment for age and gender only.^16^ When smoking was also adjusted for, risk of lung cancer mortality diminished but remained significant (odds ratio 1.73, 95% confidence interval 1.01‐2.97). However, opposing risk estimates were observed when the association was restricted to smokers (odds ratio 1.94, 95% confidence interval 1.14‐3.30) and never‐smokers (odds ratio 0.58, 95% confidence interval 0.12‐2.78). This is an indication that smoking may be a strong contributory factor in the association between periodontal disease and lung cancer. For Tu et al,^17^ however, lung cancer mortality was not associated at all with periodontal disease.

### 9.1 Newer epidemiologic evidence

Few published articles have emerged on the association between periodontal disease and lung cancer risk in recent years. Summary estimates from a meta‐analysis based on the assessment of five cohort studies associated increased lung cancer risk with the presence of periodontal disease (hazard ratio 1.24, 95% confidence interval 1.13‐1.36; *P* for heterogeneity = .22).^77^ Results from another recent meta‐analysis, also based on five cohort studies, showed a positive association between periodontal disease and lung cancer risk, pooled relative risk 1.33, 95% confidence interval 1.19‐1.49; with no statistical heterogeneity detected (*I* ^2^ = 0, *P *= 0.58).^9^ These results need to be interpreted cautiously since each meta‐analysis only included a few observational studies that adopted different methods of periodontal disease assessment. In one of the studies included in the meta‐analysis by Michaud et al,^9^ although the authors controlled for smoking status and duration, the association between periodontal disease (determined via self‐report) and lung cancer risk prevailed (hazard ratio 1.24, 95% confidence interval 1.07‐1.45). A stronger association was noted among former smokers who stopped smoking >20 years ago (hazard ratio 1.34, 95% confidence interval 1.05‐1.70), but no association was observed when this was restricted to never‐smokers (hazard ratio 1.02, 95% confidence interval 0.68‐1.53). Interestingly, there was also a lack of association with current smokers (hazard ratio 1.15, 95% confidence interval 0.81‐1.63) in a Women's Health Initiative‐Observational Study.^78^

Other published findings include a study based only on never‐smokers with 109 established lung cancer cases that showed no association between periodontal disease and lung cancer risk (hazard ratio 1.27, 95% confidence interval 0.77‐2.10).^5^ Another study did not control for smoking in their multivariate analyses and found no meaningful association between chronic periodontitis and lung cancer risk (hazard ratio 1.05, 95% confidence interval 0.98‐1.14).^11^ A third study, involving 200 Greek adults with 64 recorded cases of lung cancer, reported that probing pocket depth and bleeding on probing correlated with increased lung cancer risk after adjusting for potential confounders including smoking. When limited to nonsmokers, though, the association became insignificant.^79^ Lastly, a study published in January 2018 associated clinically measured severe periodontitis (based on the Center for Disease Control/American Academy of Periodontology definition) with a higher risk of lung and bronchus cancer (hazard ratio 2.37, 95% confidence interval 1.41‐3.99), with whites found to be slightly more at risk (hazard ratio 2.36, 95% confidence interval 1.27‐4.39) compared with blacks (hazard ratio 2.20, 95% confidence interval 0.83‐5.78). Never‐smokers were similarly affected (hazard ratio 4.24, 95% confidence interval 1.23‐14.60),^6^ but since there were too few never‐smokers, the results should be interpreted with caution.

Overall, the research evidence suggests a possible synergistic effect between periodontal disease and smoking in relation to lung cancer risk. Sensitivity analysis tests and finer adjustment of traditional confounders, particularly smoking status, may help to bring clarity to the true association between periodontal disease and risk of lung cancer incidence or mortality.

## 10 PERIODONTAL DISEASE AND BREAST CANCER RISK

There is a paucity of scientific literature on the relationship between breast cancer and periodontal disease, and results have varied. One of the older studies examined the association between tooth loss (obtained via self‐report) and the risk of cancer at 14 common sites, including breast cancer, in a Japanese population. Their study population consisted of 5240 cancer cases and 10 480 age‐ and sex‐matched noncancer controls, with breast cancer accounting for 756 cases.^3^ Loss of most teeth in the mouth had no bearing on risk of breast cancer when compared with those individuals who still had ≥21 teeth remaining (odds ratio 0.79, 95% confidence interval 0.37‐1.63; *P*_trend_ = .387). A study based on data from a Swedish Twin Registry (1963‐2004, n =* *15 333) also found no association between a periodontal disease proxy measure, tooth mobility, and breast cancer risk (hazard ratio 1.12, 95% confidence interval 0.75‐1.68).^2^ Another Swedish study (n* *=* *3273) found that more breast cancer cases were recorded among women who had periodontal disease accompanied by missing molar teeth (5.5%) compared with those women with periodontal disease and intact molar teeth (0.5%) (*P* < .02).^80^ However, almost half of the participants (n* *=* *1597) did not receive any clinical oral examination. There were 41 recorded breast cancer cases and only five of those cases involved women who received an oral clinical examination and were confirmed to have periodontal disease.

In terms of breast cancer mortality, a National Health and Nutrition Examination Survey study of 11 328 adults (1971‐1975) reported an increased but statistically insignificant risk of breast cancer mortality among those with periodontitis (hazard ratio 1.32, 95% confidence interval 0.74‐2.38).^16^

### 10.1 Newer epidemiologic evidence

A few more studies have been published on the association between periodontal disease and breast cancer since 2013. Freudenheim et al^81^ conducted the largest study to date on this association involving 2124 cases of incident invasive breast cancer based on data from the Women's Health Initiative‐Observational Study. Periodontal disease history (via self‐report) was found to be linked to an increased risk of breast cancer (hazard ratio 1.14, 95% confidence interval 1.03‐1.26) after adjustment for a number of established breast cancer risk factors, including body mass index, age at menarche, parity, age at first birth, and age at menopause. However, breast cancer risk was substantially reduced with additional adjustments for smoking status and pack‐years. Women who were former smokers who had quit within the last 20 years were particularly vulnerable (hazard ratio 1.36, 95% confidence interval 1.05‐1.77), whereas those who had never smoked were not at significant risk (hazard ratio 1.06, 95% confidence interval 0.91‐1.24). Residual confounding from smoking may have played a role in the positive associations observed. However, underreporting of periodontal disease status may have occurred from misclassification of periodontal disease status and may have resulted in the attenuation of study findings. Another study involved a subpopulation of the Women's Health Initiative‐Observational Study, the Buffalo OsteoPerio study (n =* *1337 postmenopausal women). Periodontal disease status was assessed based on radiographic analyses of alveolar crestal height. The risk of breast cancer was not related to either mild/moderate (hazard ratio 1.15, 95% confidence interval 0.67‐1.99) or severe periodontal disease (hazard ratio 0.94, 95% confidence interval 0.49‐1.82) after adjusting for age and smoking.^82^ The sample size for breast cancers in this study was small (n* *=* *89 cases), and as such there may have been inadequate power to reliably detect a small effect (if any). Likewise, Michaud et al^6^ observed that breast cancer risk was not associated with varying clinical measures of severe periodontitis (based on the Center for Disease Control/American Academy of Periodontology definition), and when limited to never‐smokers.

Findings from three other independent studies all reported a marked increase in breast cancer risk relative to periodontal disease. One was a retrospective cohort study based on 40 206 females, with equal numbers of cases and matched controls and 267 reported breast cancer cases. The rate of breast cancer was significant higher among women with chronic periodontitis than the comparison cohort without periodontitis (hazard ratio 1.23, 95% confidence interval 1.11‐1.36).^11^ It is important to note, though, that established risk factors for breast cancer and smoking status were not controlled for in multivariate analyses. In the second study, Turkish female participants with moderate/severe periodontitis showed a greater than 2‐fold increase in breast cancer relative to the expected risk for a similar age‐ and sex‐matched group (hazard ratio 2.40, 95% confidence interval 0.88‐5.33).^83^ However, the wide confidence interval observed because of the small sample size of breast cancer cases (n* *=* *5), coupled with the fact that smoking and other important risk factors were not adjusted for, implies an association cannot be readily inferred. The third study, composed of 87 breast cancer cases and 134 controls located in Brazil, utilized four different case definitions for periodontitis and found that the odds of having breast cancer in all instances varied from 2‐ to 3‐fold based on the case definition applied.^84^

In the relationship between periodontal disease and breast cancer risk, smoking status and other factors, perhaps not immediately apparent, may play a contributory role. More careful, well‐planned studies are needed to shed more light on the true nature of this association.

## 11 PERIODONTAL DISEASE AND CANCER RISK: OTHER LESS COMMON MALIGNANCIES

For a number of cancer sites, there is very little published epidemiologic data primarily because the number of documented cancer cases at these sites in individuals with periodontal disease are often too limited in number to allow for any robust statistical analyses or meaningful conclusions to be drawn. Nevertheless, some such uncommon cancer sites in relation to periodontal disease are briefly described below.

### 11.1 Gall bladder cancer

The only published study in relation to gall bladder cancer risk reported a significantly greater risk of diagnosis of gall bladder cancer (n = 60) among those who self‐reported a history of periodontal disease compared with those with no history of periodontal disease (hazard ratio 1.73, 95% confidence interval 1.01‐2.95). However, upon restriction to never‐smokers only, the association became markedly reduced (hazard ratio 1.26, 95% confidence interval 0.59‐2.68).^4^

### 11.2 Liver cancer

In 2008, a study examined periodontal disease and risk of liver cancer, with 167 recorded liver cancer cases out of 5240 cancer cases overall and 10 480 controls matched for age and sex. The investigators found no associated risk of liver cancer with varying categories of tooth loss compared with those with ≥21 remaining teeth.^3^ Their findings were similar to another study that reported no association between periodontal disease obtained via self‐report and liver cancer risk (n = 62) in a Women's Health Initiative‐Observational Study cohort (hazard ratio 1.33, 95% confidence interval 0.77‐2.29).^4^ However, another group of investigators reported a higher risk of liver cancer in individuals who had either experienced the loss of many teeth (11‐31 permanent teeth, hazard ratio 1.42, 95% confidence interval 1.01‐1.98) or were completely edentulous (hazard ratio 1.45, 95% confidence interval 1.00‐2.10) when compared with those reporting a loss of 0‐10 teeth. The study was prospective in nature and comprised 29 096 Finnish male smokers drawn from the Alpha‐Tocopherol, Beta‐Carotene Cancer Prevention study, with 213 cases of primary liver cancers.^85^ Following additional adjustments for *Helicobacter pylori* infection and established risk factors of liver cancer, hepatitis B, and hepatitis C, the risk persisted but was nonsignificant. The loss of many teeth did not result in an elevated risk of liver cancer mortality in a study involving Swedish adults (hazard ratio 1.26, 95% confidence interval 0.55‐2.90),^10^ but risk of death from liver cancer was near significance in an elderly Japanese cohort (hazard ratio 1.07, 95% confidence interval 0.98‐1.17).^56^ Only 13 deaths from liver cancer were recorded in this study.

### 11.3 Prostate cancer

Lee et al 86 studied the relationship between periodontal disease and prostate cancer risk in 187 934 South Korean adults aged ≥40 years over a 12‐year period. Presence of periodontal disease was determined from its diagnosis code based on a combination of clinical and radiographic dental records from the National Health Insurance Service‐Health Examinee Cohort. They reported an elevated risk of prostate cancer among those with periodontal disease (hazard ratio 1.14, 95% confidence interval 1.01‐1.31) after adjustment for smoking, alcohol, and sociodemographic factors.^86^ Their findings contradict those of Michaud et al,^6^ who did not find varying clinical measures of severe periodontitis (classified according to the Center for Disease Control/American Academy of Periodontology definition) to be associated with risk of prostate cancer. Similarly, an earlier study examined history of periodontal disease in relation to risk of advanced prostate cancer and reported a negative association after adjustment for pertinent factors including smoking (hazard ratio 0.89, 95% confidence interval 0.71‐1.10).^1^ Their findings, when limited to never‐smokers in a later study, were also null (hazard ratio 1.17, 95% confidence interval 0.94‐1.47).^5^ In these two latter USA‐based cohort studies, as well as a case‐control Japanese study,^3^ varying categories of tooth loss were not associated with prostate cancer risk. On the other hand, a study with tooth mobility as a proxy measure for periodontal disease reported a 47% greater risk of prostate cancer among those with periodontal disease compared with those no evidence of periodontal disease (hazard ratio 1.47, 95% confidence interval 1.04‐2.07).^2^ Regarding the risk of prostate cancer mortality, no link was observed relative to periodontal disease when measured by clinical dental examination^16^ or via records of procedural diagnostic codes.^10^

### 11.4 Hematological/hematopoietic malignancies

Few studies have associated periodontal disease with elevated risks of hematological cancers combined (hazard ratio 1.18, 95% confidence interval 1.02‐1.37),^11^ and of lymphoid/hematopoietic malignancies combined in never‐smokers (hazard ratio 1.34, 95% confidence interval 1.08‐1.67).^4^ Michaud et al^1^ reported increased risks with hematopoietic malignancies (hazard ratio 1.30, 95% confidence interval 1.11‐1.53), but in a later study published in 2018, the authors noted clinical measures of periodontal disease were not linked to hematopoietic and lymphatic cancers combined (hazard ratio 0.89, 95% confidence interval 0.52‐1.52).^6^ Other reports indicate periodontal disease is linked to non‐Hodgkin lymphomas (hazard ratio 1.30, 95% confidence interval 1.11‐1.51),^87^ but not lymphomas in general (hazard ratio 1.17, 95% confidence interval 0.40‐3.44),^3^ or leukemias (hazard ratio 1.10, 95% confidence interval 0.83‐1.47).^4^

### 11.5 Genitourinary cancers

Periodontal disease has been linked to an elevated risk of genitourinary cancers combined (hazard ratio 1.30, 95% confidence interval 1.21‐1.39).^11^ No associations were observed with cancers of the urinary tract system (hazard ratio 1.16, 95% confidence interval 0.92‐1.45),^4^ or bladder (hazard ratio 1.17, 95% confidence interval 0.96‐1.43),^1^ (hazard ratio 2.85, 95% confidence interval 0.57‐14.22),^3^ (hazard ratio 1.13, 95% confidence interval 0.59‐2.20).^2^ A significantly elevated risk has been reported in relation to kidney cancer (hazard ratio 1.49, 95% confidence interval 11.12‐1.97),^1^ but not when limited to never‐smokers alone (hazard ratio 1.06, 95% confidence interval 0.61‐1.85).^1^ Although a positive association has been noted with uterine cancer (hazard ratio 2.20, 95% confidence interval 1.16‐4.18),^2^ negative associations have been reported with risks of cancers of the female genital organs combined (hazard ratio 1.10, 95% confidence interval 0.95‐1.29),^4^ ovary (hazard ratio 0.18, 95% confidence interval 0.02‐1.55),^3^ (hazard ratio 0.86, 95% confidence interval 0.64‐1.15),^88^ and uterus (hazard ratio 0.90, 95% confidence interval 0.43‐1.88).^3^

Studies on cancers rarely reported in association with periodontal diseases have been mostly negative and include cancers of the thyroid (hazard ratio 1.27, 95% confidence interval 0.38‐4.25),^3^ and brain (hazard ratio 0.99, 95% confidence interval 0.61‐1.59).^1^ A positive association with melanoma skin cancers has been reported (hazard ratio 1.23, 95% confidence interval 1.02‐1.48),^4^ although another group of investigators observed no such link (hazard ratio 1.06, 95% confidence interval 0.86‐1.30),^1^ even when limited to never‐smokers (hazard ratio 1.20, 95% confidence interval 0.89‐1.63).^5^

### 11.6 Periodontal disease treatment and cancer risk

No randomized controlled clinical trials on periodontal disease treatment and cancer risk exist but a few association studies have been published. One such study investigated the effects of routine treatment of periodontal disease on cancer risk using data from the Taiwan National Health Insurance system. The treatment cohort comprised 38 902 participants who had been diagnosed with periodontal disease and received at least 10 treatments, including subgingval curettage (scaling and root planing), and periodontal flap surgery. The comparison cohort consisted of 77 804 participants. Two age‐ and sex‐matched individuals with no recorded evidence of treatment of periodontal disease were randomly selected for each treatment cohort member.^12^ The periodontal disease treatment cohort showed a marked reduction in overall cancer risk (hazard ratio 0.72, 95% confidence interval 0.68‐0.76) relative to the comparison cohort. Risk of cancer of the esophagus (hazard ratio 0.20, 95% confidence interval 0.12‐0.34), colon/rectum (hazard ratio 0.70, 95% confidence interval 0.60‐0.82), lung (hazard ratio 0.45, 95% confidence interval 0.38‐0.54), female reproductive tract (hazard ratio 0.58, 95% confidence interval 0.46‐0.72), and brain (hazard ratio 0.35, 95% confidence interval 0.18‐0.67) were similarly reduced. Conversely, risks of cancers of the prostate (hazard ratio 2.11, 95% confidence interval 1.63‐2.73) and thyroid (hazard ratio 1.54, 95% confidence interval 1.09‐2.09) were significantly elevated in the treatment cohort. There was a lack of adjustments for pertinent factors such as smoking status, alcohol consumption, and other lifestyle/behavioral characteristics. Study participants reported higher frequencies of undergoing thyroid diagnostic procedures and prostate‐specific antigen testing, which may have contributed to the elevated risks in prostate and thyroid cancers observed. Furthermore, the periodontal disease prevalence rate among this group was unusually high at 95%, thus the findings may not be applicable to other study populations.

A retrospective study drawn from a database of 718 409 Taiwanese individuals (periodontal disease, n = 519 831; esophageal carcinoma, n* *=* *682), observed a significantly reduced risk of esophageal carcinoma among male participants with periodontal disease who received dental prophylaxis (hazard ratio 0.54, 95% confidence interval 0.44‐0.66) compared with those without periodontal disease. No difference was noted among a similar group of females (hazard ratio 0.62, 95% confidence interval 0.31‐1.23), individuals with periodontal disease who received intensive treatment (hazard ratio 0.96, 95% confidence interval 0.78‐1.18), or those who had no treatment at all (hazard ratio 1.27, 95% confidence interval 0.89‐1.82).^13^ Patients who received intensive treatment probably had severe periodontal disease. There was no indication if clinical resolution of periodontal disease was achieved, or regarding the timing of recorded results among the treatment groups. In addition, important risk factors for esophageal cancer, such as family history, body mass index, and smoking status, were not taken into consideration in the analysis. These factors could potentially have influenced the results that were obtained.

In other studies, Moergel et al^14^ reported that history of periodontal treatment is inversely associated with oral cancer risk (odds ratio 0.2, 95% confidence interval 0.1‐0.5). Similarly, Chung et al^11^ found that the risk of cancer increased significantly among participants with chronic periodontitis who did not receive any periodontal treatment compared with those who did not have chronic periodontitis (hazard ratio 1.29, 95% confidence interval 1.21‐1.32). However, the authors did not observe any significant differences in cancer risk between participants with chronic periodontitis who received periodontal treatment (gingivectomy or periodontal flap operation) relative to the comparison cohort without periodontal disease (hazard ratio 1.17, 95% confidence interval 0.86‐1.58). As such, the authors surmised that lack of periodontal treatment, and not the presence of the disease itself, may influence cancer risk.

Since the evidence on periodontal disease treatment and risk of cancer has been based on observational studies and not from randomized controlled clinical trials, such evidence is subject to the same biases and issues relating to confounder adjustments typically observed with the former, and so no firm conclusions on causality or periodontal disease management can be inferred from these findings.

## 12 PLAUSIBLE MECHANISTIC LINKS BETWEEN PERIODONTAL DISEASE AND CANCER RISK

The mechanism through which cancer may develop among individuals who have periodontal disease is not entirely clear. A number of plausible mechanisms have been proposed and inflammation appears to play a significant role in many of these postulated mechanisms. This is not surprising as periodontal disease is a prototype of an infectious process that induces chronic low‐grade inflammation if left untreated. Infection has been known to promote inflammation, and persistent low‐grade inflammation has been linked to cancer.^89^, ^90^, ^91^ Inflammation has been identified to act as a crucial enabler to the six widely recognized biological capabilities necessary for malignant change to occur (ie, the hallmarks of cancer).^92^ Inflammatory processes can generate free radicals and active intermediates causing oxidative/nitrosative stress, which may lead to DNA mutations in cells, or they may interfere with DNA repair mechanisms.^93^ The inflammatory cells themselves may further contribute to the damage by producing free radicals, cytokines, chemokines, and metabolites of arachidonic acid; the generated products, in turn, demonstrate a strong affinity for more inflammatory cells, perpetuating the vicious cycle.^93^

Studies have also shown that periodontal therapy can substantially decrease markers of systemic inflammation,^94^, ^95^ and that certain anti‐inflammatory drugs may help prevent or decrease the risk of certain site‐specific cancers, including those of the colorectum, esophagus, stomach, biliary tract, and breast.^96^, ^97^ As a result, some newer epidemiologic studies are investigating the role of oral microbiome and/or specific established periodontal pathogens in relation to periodontal disease and cancer risk.

### 12.1 Oral microbiome and inflammation in relation to cancer risk

In periodontitis, subgingival biofilms serve as reservoirs of anaerobic, gram‐negative bacteria.^98^ Periodontal pathogens such as *Porphyromonas gingivalis* and *Aggregatibacter actinomycetemcomitans* release enzymes that aid in digesting extracellular matrix components including collagen in order to produce substrates for their own nutrition and to enhance tissue invasion.^99^ These bacterial enzymes, as well as other bacterial components such as endotoxins, and metabolic by‐products that are naturally toxic to tissues, may cause direct DNA damage to neighboring epithelial cells. They can induce mutations in proto‐oncogenes and tumor suppressor genes, or interfere with the molecular pathways involved in cell proliferation and/or survival.^100^

Notable differences have been observed in the composition of the bacterial microflora of tumor tissue within the oral cavity compared with nontumor sites, and this shift in bacterial colonization may be associated with an increased risk of oral squamous cell carcinomas.^101^ Oral bacteria may promote oral carcinogenesis by constitutively activating toll‐like receptors (such as toll‐like receptor‐5).^102^ Toll‐like receptors usually present on the surfaces of cells of the innate immune system have also been associated with epithelial and cancer cells,^103^ and are implicated in inflammation, cellular proliferation, invasion, and evasion of antitumoral immune responses.^104^, ^105^

Periodontal pathogens such as *Fusobacterium nucleatum* have been isolated from inflammatory bowel disease conditions, including Crohn's disease^106^ and ulcerative colitis.^107^ They have also been detected in premalignant lesions such as colorectal adenomas^108^ and colorectal cancers.^109^, ^110^ Kostic et al^109^ reported the presence of *F. nucleatum* DNA sequences in much greater quantities among their nine human colorectal cancer tissue samples compared with their matched controls of normal colon. Rubinstein et al^111^ demonstrated that *F. nucleatum* can adhere to, and penetrate, colonic tissue where it may upregulate local inflammatory responses and promote growth factors that favor the proliferation of colorectal cancer cells via its FadA adhesion properties. Kostic et al^108^ used mouse models (Apc [Min/+] mice) to demonstrate that *F. nucleatum* can specifically attract tumor‐infiltrating myeloid cells and create a proinflammatory milieu that promotes colorectal carcinogenesis. Another line of investigation by this group showed that *F. nucleatum* potentiates intestinal tumorigenesis by modulating the antitumor immune system.^108^ *Fusobacterium nucleatum* were shown to expand myeloid‐derived immune cell types such as Fox‐alpha3 and T‐reg cells that promote tumor progression by suppressing cytotoxic and effector T cells, thus diminishing local antitumor immunity. Furthermore, the effects on antitumor immunity were specific for *Fusobacterium*, as they were not seen with *Escherichia*,* Streptococcus*, or *Propionibacterium*, which are highly abundant genera in the gut microbiome. *Fusobacterium nucleatum*, in combination with *P. gingivalis*, have been reported to promote carcinogenesis by upregulating the interleukin‐6/signal transducer and activator of transcription 3 (IL‐6/STAT3) pathway via the activation of toll‐like receptors on oral epithelial cells.^112^ Much has to be learned about the relative importance of *F. nucleatum* compared to genetic and environmental factors also operative in initiating and promoting the spread of colorectal cancer. However, it is encouraging that there is strong evidence for a key role of *F. nucleatum* since this organism may be amenable to control with present antimicrobial therapies. These findings suggest that periodontal disease may be causally linked to colorectal cancer, and therefore the association should be examined in greater detail.

Peters et al^113^ have shown that the periodontal pathogen *Tannerella forsythia* in the oral microbiota is associated with esophageal adenocarcinoma and depletion of the oral microbiome of the commensal *Neisseria* genus, and that *Streptococcus pneumoniae* were associated with a lower risk of esophageal adenocarcinoma. They also found that the abundance of *P. gingivalis* trended with a higher risk of esophageal squamous cell carcinoma. These results are consistent with the findings of Gao et al,^114^ who reported that *P. gingivalis* was found in 61% of their samples of esophageal tissues from patients with esophageal squamous cell carcinoma, but was not detected in normal esophageal mucosa. These findings suggest an association between infection with periodontal pathogens and esophageal cancers, which is consistent with the epidemiology findings of Nwizu et al.^4^ A detailed review of epidemiologic studies relating to the human microbiome and cancer risk has been elaborated on elsewhere.^115^, ^116^

### 12.2 Periodontal disease and cancer risk – the oral‐systemic link

Dense collections of gram‐negative anaerobic bacteria that have been detected in the periodontal pockets of people with periodontitis^117^ may become detached and be micro‐aspirated or ingested. Alternatively, the ulcerated periodontal pocket walls may unwittingly serve as a potential avenue for the escape of toxic metabolites,^34^ or the oral bacteria themselves and their associated components, into the systemic circulation, to lodge at distant body sites.^118^ This proposed mechanism is supported by the fact that transient bacteremia has been reported following routine periodontal examination,^119^ and during daily activities such as toothbrushing, and may occur more frequently among individuals with gingival inflammation.^120^ Masticatory forces also appear to enhance the release of significant amounts of oral bacterial proinflammatory components such as lipopolysaccharides into the systemic circulation in individuals with severe periodontitis.^121^ Alhough the release of these oral bacteria into the blood stream is temporary, periodontal pathogens such as *P. gingivalis* can evade their uptake and destruction by phagocytes via their protease production.^122^ *Porphyromonas gingivalis* can further circumvent their destruction by modulating Th_2_‐cell‐mediated, anti‐inflammatory responses to favor M_2_ macrophage phenotype production, whih are less capable of eliminating them.^123^, ^124^ It has been shown that dendritic cells phagocytose but do not destroy *P. gingivalis* microorganisms, thus allowing these intracellular bacterial cells to reach distant organs.^125^ These inherent biologic properties of *P. gingivalis* could potentially enable them to survive within the systemic circulation and remain viable enough to reach remote body sites and produce deleterious effects.

This theory is supported in part by the fact that periodontal pathogens have been isolated from many parts of the body besides the oral cavity including atheromatous plaques of blood vessels,^126^, ^127^, ^128^ lymph nodes,^129^ lung aspirates,^130^, ^131^ pericardial fluid,^132^ liver tissues,^133^ spinal infections,^134^ tonsils,^135^ and the appendix.^136^ It is especially important to note that oral organisms have also been found in precancerous lesions of the stomach^137^ and of the colon (ie, colorectal adenomas),^108^ and certain cancers such as colorectal carcinomas,^109^, ^110^ oral carcinomas,^138^ esophageal, and gastric cancers.^139^, ^140^

Oral bacteria in the bloodstream may cause harm via their lipopolysaccharide component, by inducing either a systemic^141^ or local inflammatory response at the site where they lodge. Lipopolysaccharide is a prototype of a pathogen‐associated molecular pattern^142^ and a potent inducer of inflammation, even in tiny amounts.^143^, ^144^ Pathogen‐associated molecular patterns serve as ligands for toll‐like receptors present on cell surfaces of the innate immune system known to instigate inflammation.^142^

Okuda et al^145^ used mouse models to isolate periodontal pathogens in bronchoalveolar lavage fluids and demonstrate their link to the development of local inflammation such as pneumonia. In a similar fashion, using data based on the National Health and Nutrition Examination Survey I study, Scannapieco et al^146^ found that poor oral hygiene was linked to chronic respiratory disease (n =* *23 808; cases of chronic respiratory disease, n =* *386). They posited that inflammatory mediators from the diseased periodontium could be aspirated into the lungs to promote bacterial pneumonia.^147^, ^148^, ^149^ Local inflammatory mediators produced in response to periodontal disease such as interleukin‐6, tumor necrosis factor‐alpha, and prostaglandin E_2_, escape through the damaged periodontal tissue pockets into the systemic circulation to produce their systemic effects at remote body sites.^150^

Loos et al^150^ postulated that upon passage into the bloodstream, local immune mediators activated as a consequence of periodontal infection may stimulate hepatocytes in the liver to produce copious amounts of acute‐phase proteins such as serum C‐reactive protein (an established biomarker of systemic inflammation) and other systemic inflammatory mediators. This is supported by the fact that several studies have associated increased levels of serum C‐reactive protein with periodontal disease.^150^, ^151^, ^152^, ^153^, ^154^ Some studies have also reported a positive association with individual periodontal pathogens.^152^, ^155^, ^156^, ^157^ Most studies have, however, involved relatively small study populations (<200), although there have been a few large studies.^151^, ^153^, ^158^ Furthermore, increased serum C‐reactive protein levels have been linked to increased risk of precursor lesions,^159^ as well as various cancers.^160^, ^161^ If the periodontal disease is untreated, persistence of the initiating factors is likely to occur and inflammation may fail to resolve. Over time, the chronic stimulus from the diseased periodontium could lead to the generation of persistent, low‐grade inflammation that may contribute to the carcinogenic process.^80^, ^162^ This is a likely mechanism through which periodontal disease may be linked to an increased risk of cancer.

### 12.3 Other plausible mechanistic links between periodontal disease and cancer

Refractory periodontitis has been linked to phenotypic changes in the mononuclear cell‐cytokine system resulting in a much stronger inflammatory response than usual upon exposure to certain bacterial stimuli such as lipopolysaccharide.^163^ Genetic polymorphisms involving inflammatory cytokines may contribute to individual susceptibility to disease severity^90^ and possibly cancer. Certain individuals with chronic periodontitis also possess an inherent defect in their immune system, particularly with regard to bacterial clearance and tumor immune surveillance.^164^ This may increase their susceptibility to cancer. Matrix metalloproteinases play a crucial role in extracellular matrix and basement membrane degradation.^165^ This property aids in periodontal tissue destruction,^166^, ^167^ as well as in cancer progression and metastasis, by causing tissue dissolution enabling tumor invasion.^168^, ^169^ Susceptibility to chronic periodontitis has been demonstrated among Chinese with certain matrix metalloproteinase genetic polymorphisms.^170^ This enhanced feature of matrix metalloproteinases may have implications for increased cancer risk, too.

In hyperglycemic states, as observed with diabetic patients, nonenzymatic glycation and oxidation of proteins and lipids occurs, leading to the formation of advanced glycation end products (advanced glycation end products). These substances can accumulate and cause pathogenic changes through interaction with their associated receptor, receptor for advanced glycation end products. Advanced glycation end product receptor is expressed on various cell surfaces and has been implicated in several disease conditions including inflammation, periodontal disease, diabetes, and cancer.^171^, ^172^ Advanced glycation end product‐advanced glycation end product receptor interactions can upregulate certain inflammatory cytokines like tumor necrosis factor‐alpha and other inflammatory mediators such as lipopolysaccharides, leading to overresponsive immune responses in the diabetic patient, thereby promoting bone destruction, such as observed in severe periodontal disease.^172^ Advanced glycation end product receptor ligands are also secreted by cancer cells and help to promote carcinogenesis by stimulating cancer cells directly, causing them to act autonomously. They also modulate various cell types within the tumor microenvironment, such as fibroblasts, leukocytes, and vascular cells, resulting in increased fibrosis, inflammation, and angiogenesis.^171^ Therefore, the interrelationships between periodontal disease, diabetes, and cancer risk should be given further consideration.

Advanced periodontal disease is often accompanied by tooth loss. When severe, it may reduce masticatory efficiency and lead to the avoidance of chewing tough foods like fruits and fibrous vegetables.^173^, ^174^ Unfortunately, these foods are often more nutritious and rich in cancer‐fighting agents such as antioxidants. Studies have shown that fruits and nonstarchy vegetables are associated with a decreased risk of certain cancers (oral cavity, pharynx, esophagus, stomach, and lung).^175^ Soft diets are less nutritious but more energy‐dense. High calorific diets are described as proinflammatory because they are associated with higher levels of C‐reactive protein and interleukin‐6.^176^ Diets with higher dietary inflammatory index scores may promote cancer risk.^177^, ^178^, ^179^ Finally, epigenetic changes resulting in the hypermethylation of E‐cadherin and cyclooxygenase‐2 have been associated with chronic periodontitis, and may be linked with increased cancer risk.^180^

## 13 CONCLUSIONS

Our understanding of the relationship between periodontal disease and the risk of developing certain cancers is still evolving. Available data from epidemiologic evidence on periodontal disease and cancer risk mostly points to a positive association. It appears that risk may be higher for certain anatomic sites, particularly those in close proximity to the oral cavity (esophagus, upper gastrointenstinal tract). However, proving the unequivocal presence of an association between periodontal disease and cancer risk remains a difficult challenge. The majority of epidemiologic data have been derived from observational studies with attendant difficulties relating to the use of diverse proxy measures for periodontal disease ascertainment. Other challenges include issues with residual confounding by smoking in study populations involving smokers, and proper accountability of other unanticipated extraneous factors that might contribute to the association. There is therefore a need for further exploration using randomized controlled clinical trials and well‐designed, large prospective studies to help clarify the nature of association and, in particular, add evidence to support causality between periodontal disease and risk of cancer. These studies should strive to incorporate standardized clinical measurements in ascertaining periodontal disease status. More investigation is also needed to assess how improved periodontal disease prevention and management strategies may impact cancer risk.

Ever since the Joint European Federation of Periodontology/American Academy of Periodontology Workshop on “Periodontitis and Systemic Diseases” in November 2012, several other studies have added to the evidence that could support biological plausibility. The role of periodontal disease and oral microorganisms needs to be studied further, including how they are involved in the association between periodontal disease and cancer risk. Such studies may lead to novel approaches to prevent some cancers. Since there is also some evidence that indicates inflammation may mediate the association between periodontal disease and/or its pathogens and cancer risk, more research may help gain a better understanding in this regard.
